# Androgenic anabolic steroid-induced liver injury: two case reports assessed for causality by the updated Roussel Uclaf Causality Assessment Method (RUCAM) score and a comprehensive review of the literature

**DOI:** 10.1136/bmjgast-2020-000549

**Published:** 2020-11-19

**Authors:** Robin Daniel Abeles, Matthew Foxton, Shahid Khan, Robert Goldin, Belinda Smith, Mark R Thursz, Suman Verma

**Affiliations:** 1 Divison of Surgery and Cancer, Department of Digestive Diseases, St Mary's Hospital, Imperial College London, London, UK; 2 Department of Hepatology, Chelsea and Westminster Healthcare NHS Trust, London, UK; 3 Centre for Pathology, St Mary's Hospital, Imperial College, London, UK

**Keywords:** acute hepatitis, drug induced hepatotoxicity, drug induced liver injury, liver biopsy

## Abstract

**Background:**

Anabolic androgenic steroids (AAS) usage is widespread and increasing. AAS drug-induced liver injury (DILI) is recognised but its clinical course and management is poorly described. We report 2 cases of AAS DILI with associated renal dysfunction, managed successfully with oral corticosteroids.

**Methods:**

A comprehensive review identified 50 further cases to characterise the clinical and biochemical course. Causality grading was calculated using the updated Roussel Uclaf Causality Assessment Method (RUCAM) score. Data are presented as median values.

**Results:**

The most common AAS taken was methyldrostanolone. Patients commonly present with jaundice and pruritus but may exhibit other constitutional symptoms. Patients presented 56 days after starting, and bilirubin peaked 28 days after stopping, AAS. Causality assessment was ‘unlikely’ in 1 (2%), ‘possible’ in 31 (60%) and ‘probable’ in 20 (38%). Peak values were: bilirubin 705 μmol/L, alanine transaminase 125 U/L, aspartate transaminase 71 U/L, alkaline phosphatase 262 U/L, gamma-glutamyl transferase 52 U/L, international normalised ratio 1.1. Liver biopsies showed ‘bland’ canalicular cholestasis. 43% of patients developed kidney injury (peak creatinine 225 μmol/L). Therapies included antipruritics, ursodeoxycholic acid and corticosteroids. No patients died or required liver transplantation.

**Conclusions:**

Physicians are likely to encounter AAS DILI. Causality assessment using the updated RUCAM should be performed but defining indications and proving efficacy for therapies remains challenging.

## Introduction

Anabolic androgenic steroid (AAS) use for performance enhancing and cosmetic reasons is rising with a lifetime prevalence of 3%–4% in Europe and the USA.^1^

Although the potential for cholestatic AAS drug-induced liver injury (DILI) has been recognised for many years,^2^ the clinical course and optimal management of these patients remains unclear. We present two cases of AAS DILI and perform the most comprehensive literature review to date of the topic.

## Case 1

A man aged 30 years presented with a short history of jaundice and diarrhoea. He had no significant risk factors for chronic liver disease. He used Creatine supplements for performance enhancement but denied AAS use. Physical examination was unremarkable besides jaundice. His bilirubin was 181 μmol/L, alkaline phosphatase (ALP) 66 IU/L, alanine transaminase (ALT) 257 IU/L and creatinine (Cr) 97 μmol/L. Imaging ruled out biliary or vascular abnormalities. A liver screen was taken and urgent follow-up organised.

He represented with worsening lethargy and malaise 7 days later. His bilirubin had risen to 373 μmol/L but all other tests were stable. His liver screen identified undiagnosed chronic hepatitis B (HBV) with a low viral load (181 IU/mL) and a low titre of antismooth muscle antibody (1:40) but nothing else suggestive of autoimmune hepatitis. However, he was started on prednisolone 60 mg at the referring hospital with tenofovir prophylaxis against HBV ‘reactivation’. On arrival to our hospital, his bilirubin had risen to 526 μmol/L, his international normalised ratio (INR) remained normal and Cr peaked at 126 μmol/L. Repeat autoimmune screen was negative and HBV DNA was fully suppressed (<20 IU/mL).

On repeat questioning he admitted to taking methyldrostanolone, starting 6 weeks, and finishing 2 weeks, prior to presentation.

**Figure 1:**
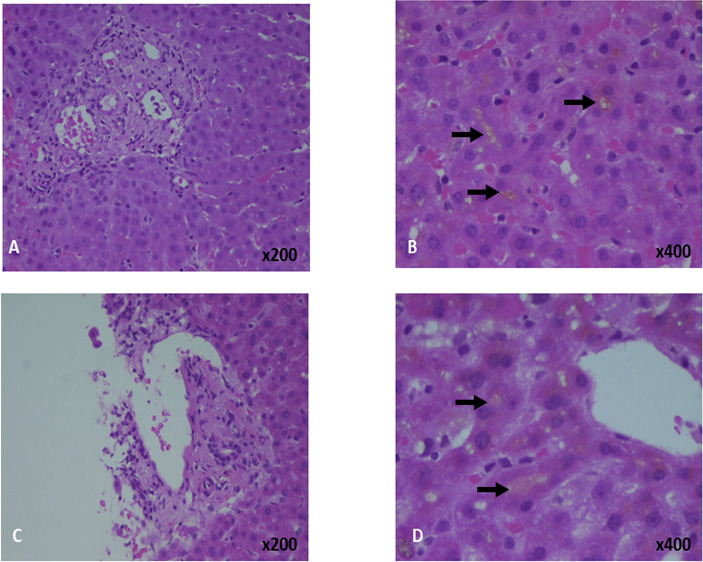
Liver biopsies from patient 1 (A, B) and patient 2 (C. D). The biopsies show no significant inflammation (A, C). On higher power magnification, canalicular cholestasis with bile plugs is demonstrated (arrows).

Intravenous N-acetyl cysteine (NAC) was given, stopping 48 hours later along with prednisolone. His bilirubin dropped to 486 μmol/L but rose again to 633 μmol/L. Other peak values included ALT 257 IU/L, aspartate transaminase (AST) 143 IU/L, ALP 123 IU/L, gamma-glutamyl transferase (GGT) 31 IU/L and INR 1.1. As his liver biochemistry had worsened, he proceeded to liver biopsy to ensure no alternative pathology was present. The biopsy showed fibrous expansion of the portal tracts, reactive ductular changes with only mild-to-moderate inflammation without plasma cells or eosinophils but marked canalicular cholestasis (figure 1A, B).

AAS DILI was suspected with an R score of 11.7, consistent with hepatocellular pattern. The updated Roussel Uclaf Causality Assessment Method (RUCAM) score was 4 consistent with ‘possible’ causality.^3^

**Figure 2:**
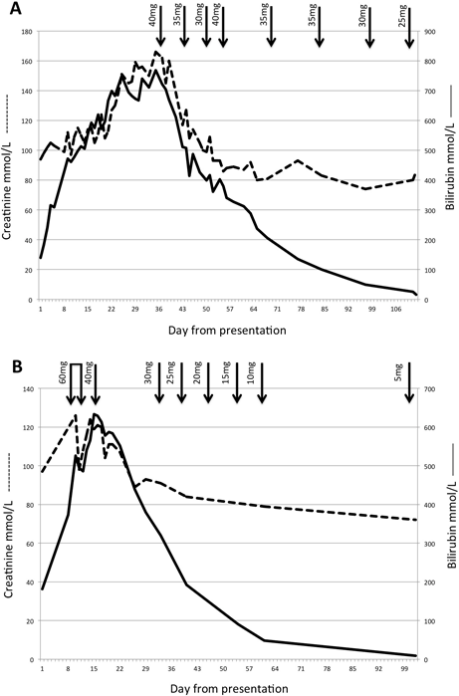
Changes in bilirubin (μmol/L, solid line) and creatinine (μmol/L, dashed line) from day of presentation in patient 1 (A) and patient 2 (B). Arrows indicate dose of prednisolone.

Prednisolone 40 mg was restarted with a temporally related drop in bilirubin and Cr and he was discharged 4 days later. Liver function tests resolved (ALT 61 IU/L, ALP 56 IU/L, bilirubin 9 μmol/L) over 2 months as the steroids were tapered down and stopped (figure 2A).

## Case 2

A man aged 36 years presented with non-specific abdominal pains followed by 5 days of jaundice and pruritus. He had no risk factors for chronic liver disease. He admitted to using Creatine and ‘Winter Cherry’ (Ashwagandha) for 2 months, stopping 1 month prior to presentation, for performance enhancement. On further questioning, he then admitted to also having taken methyldrostanolone.

He was jaundiced with excoriations but no peripheral stigmata of chronic liver disease. His bilirubin was 140 μmol/L, ALT 460 IU/L, ALP 219 IU/L, GGT 25 IU/L, Cr 94 μmol/L and INR 1.0. His liver screen was unremarkable. Imaging excluded biliary or vascular pathology. He was started on chlorphenamine and ursodeoxycholic acid (UDCA) for pruritus. Despite 5 days of intravenous NAC, his bilirubin rose to 428 μmol/L. A liver biopsy was performed due to this rise to exclude alternative pathology. The biopsy showed mild focal lymphocytic infiltration with marked canalicular and intrahepatocyte cholestasis (figure 1C, D).

Possible AAS DILI was diagnosed with an R score of 6.3 (hepatocellular) and a RUCAM score of 4.^3^ Despite increasing the UDCA and changing to hyroxyzine, his bilirubin rose to 750 μmol/L and Cr to 151 μmol/L. He was given empirical antibiotics and intravenous fluids in case of covert infection but despite a transient improvement, the bilirubin rose again peaking at 768 μmol/L associated with a Cr of 166 μmol/L. Prednisolone 40 mg was started with an improvement in bilirubin and Cr over the next 7 days. The prednisolone was weaned rapidly to 25 mg but the bilirubin plateaued at ~400 μmol/L. Prednisolone was increased to 40 mg, associated with a further improvement in bilirubin and normalisation of Cr. The patient was discharged 3 days later and his prednisolone was weaned slowly (figure 2B). The bilirubin, ALT and ALP normalised over the next 2.5 months (bilirubin 16 μmol/L, ALT 73 IU/mL, ALP 123 IU/L).

## Review of the published literature

### Methods

A PubMed search using the terms ‘anabolic steroid liver’ returned 966 titles. Titles and abstracts were screened for cholestatic AAS DILI. Non-English or non full-text articles were excluded. Included manuscripts’ references were also screened for relevance. Thirty manuscripts were included in the final analysis.

The largest published single series (Spanish-South American registry) comprised 25 patients,^4 5^ however, only summary statistics were provided so individual cases could not be included but serve as a comparison group. All data are presented as median and IQR.

The R value, updated RUCAM score and the causality grading was calculated for each case.^3^

### Results

Fifty-two patients (50 male, median age 29 (IQR 25–41)) were included (online supplemental table 1). Besides the earliest cases,^6 7^ AAS were taken for performance enhancement. The most common AAS, either alone or in combination was methyldrostanolone (methasterone). Jaundice and pruritus were the most common presenting symptoms, accompanied by constitutional symptoms such as lethargy, gastrointestinal symptoms or weight loss. Median time to presentation was 56 days (IQR 42–72). At presentation Bilirubin was 314 μmol/L (IQR 174–590), ALT 125 IU/L (IQR 85–233), AST 71 IU/L (IQR 58–112) and ALP 190 IU/L (IQR 123–287). Bilirubin peaked 28 days (IQR 14–35) after stopping AAS at 705 μmol/L (IQR 549–872). Peak ALT was 125 IU/L (IQR 85–233), AST 71 IU/L (IQR 58–112), ALP 262 IU/L (IQR 183–372), GGT 52 IU/L (IQR 29–67) and INR 1.1 (IQR 1–1.3). The time to resolution from peak bilirubin was 90 days (IQR 75–120). One patient developed encephalopathy and two developed INR ≥1.5.

10.1136/bmjgast-2020-000549.supp1 Supplementary data

The median R value was 2 (0.8–4.3) with 10 (19%) having ‘hepatocellular’ and 42 (81%) ‘mixed or cholestatic’ liver injury. The median updated RUCAM score was 5 (4–6) with causality assessments of ‘unlikely’ in 1 (2%), ‘possible’ in 31 (60%), ‘probable’ in 20 (38%). In 13 (25%) cases, concomitant bodybuilding supplements were recorded, 2 along with Ashwagahanda.

Liver biopsies predominantly showed marked canalicular and intrahepatocyte cholestasis with only mild or moderate inflammation. Twenty-two patients (43%) developed acute kidney injury (AKI) with a peak Cr of 225 μmol/L (IQR 174–406). Two patients received renal replacement therapy,^8^ one peritoneal dialysis^7^ and three molecular adsorbent recirculating system (MARS).^9^ Besides lower ALT values and higher incidences of renal dysfunction, these results are similar to those seen in the Spanish-South American series^5^ (online supplemental table 2).

10.1136/bmjgast-2020-000549.supp2 Supplementary data

‘Standard medical therapy’ or no therapeutic details were given for 24 patients.^9–18^ Seventeen patients received antipruritic therapy, predominantly antihistamines and rifampicin, colestyramine, naltrexone and phenobarbital. Twelve patients received UDCA alone, 3 received corticosteroids alone (Abeles RDA)^19 20^ and 12 received corticosteroids and UDCA (3 due to ‘failure’ of UDCA (Abeles RDA)),^19 21^ 6 patients received MARS^9 16 22^ and 1 received plasmapheresis.^23^

## Discussion

Most DILIs resolve with cessation of the causative agent. Registry data, representing the most severe forms of DILI, show mortality/liver transplant rates of 4%–10%.^24–26^ Within the US-DILIN group, dietary supplements were the primary implicating agent in 16% of those patients but the proportion due to AAS is not given.^27^ From 1963 to 2014, UK MHRA data recorded 4/61 fatal liver deaths from AAS DILI (2 related to jaundice, 1 liver failure and 1 from cirrhosis).^28^

Hy’s law predicts a 10% mortality in DILI if the transaminases are 3× upper limit of normal (ULN) and the bilirubin (excluding unconjugated hyperbilirubinaemia) is >2× ULN without initial elevated ALP.^29^ When validated in registry data studies,^25 26 30 31^ Hy’s law shows a high sensitivity but low specificity and so is used as a signal of serious hepatotoxicity in drug development rather than a clinical predictor of severe outcome. In this series, when able to calculate, 50% (17/34) fulfilled Hy’s law yet there were no deaths nor transplantations nor were there any in the Spanish-South American series.^5^ These data reassure the physician that the prognosis for AAS DILI is excellent.

The RUCAM scale (and its update) is validated and specifically designed for DILI with scores given for defined key elements to provide a causality grading assessment and has been widely used for over 25 years. It is intended to be used prospectively, raising methodological challenges when applied to historical case series with incomplete data, as in the presented work. Liver injury assessment, demonstrating ALT of at least 5× ULN and/or hepatic ALP of at least 2× ULN, is a prerequisite. Within the data available for this series, 30 (58%) fulfilled this prerequisite, rising to 35 (67%) if peak values were used instead of admission ones. This raises the possibility that despite publication, some of these cases may have been misclassified as AAS-induced DILI.

Liver biopsy is not required for diagnosis of DILI,^3^ but is often performed due to diagnostic uncertainty, especially when therapies are being considered or in the face of worsening biochemistry despite cessation of the implicating drug, as in the two cases presented. Although safe, liver biopsy carries established risks. These data, demonstrating that peak bilirubin is seen 28 days after presentation, reassure the physician that biopsy can usually be avoided. Biopsies from patients with hepatocellular injury, as defined by the R score, often have a ‘bland’ cholestatic pattern histologically, this is likely due to timing due to the evolution of a cholestatic phase after the initial hepatocellular phase. The recommendation for causality assessment is that the R value should be calculated at the first time point that qualifies as being indicative of DILI.^3^

Another challenge for causality assessment for AAS is that many patients use concomitant bodybuilding supplements, stopping concurrently, as was the case for both our presented cases. To distinguish between them, RUCAM assessment should be performed for each potential agent, thereby reducing the ‘concomitant drug/herb’ score for both agents. Hepatotoxicity is labelled on AAS leading to a higher score than supplements, so are usually favoured as the culprit agent.

Although a new diagnosis of HBV was found in case 1, this was not felt to be clinically related to his acute presentation so did not lose a point within the ‘alternative causes’ for liver disease RUCAM domain. There are conflicting data as to whether HBV affects the risk of DILI with antituberculosis medications,^32 33^ but pre-existing chronic liver disease was not shown to affect risk within the DILIN prospective study.^24^

The use of therapies such as steroids, UDCA or dialysis devices may mask the natural course of ALT or ALP in the dechallenge phase and therefore, if given, score the patient zero in the ‘course after cessation of drug’ RUCAM component.^3^ Thirty-one (60%) cases received at least one form of therapy over the dechallange phase. However, this resulted in only three patients being ‘classified down’ from ‘probable’ to ‘possible’ and one from ‘possible’ to ‘unlikely’. The presented 'case 2' was treated with UDCA with the resultant RUCAM score 3 points lower (4 vs 7) than it might have been, moving from a causality assessment of ‘probable’ to ‘possible’. In practice, the physician may have to sacrifice a degree of diagnostic rigour when faced with a decision whether to commence treatment for a patient.

As all data derive from uncontrolled case reports, ascertaining the efficacy of therapies in AAS DILI is challenging. Despite the likely publication bias favouring intervention, most reported cases of AAS DILI resolve spontaneously with no specific therapy. Although Wree *et al* found that, compared with historical controls, corticosteroid-UDCA combination resulted in a quicker reduction in bilirubin in DILI, subgroup analysis for AAS DILI was not significant.^34^ Several patients in this series progressed to second-line therapy (corticosteroids, MARS or plasmapheresis) due to lack of response of UDCA.

For our cases, a temporal relationship was seen between starting corticosteroids (or escalating after a rapid wean for patient 2) and a reduction in serum bilirubin (figure 2A, B) with no significant side effects or adrenal suppression.

The role of NAC in non-paracetamol DILI is unclear. In some cases, labelled ‘non-paracetamol ALF’, paracetamol may be the underlying aetiology.^35^ The risks of NAC are few and it may benefit in improving transplant-free survival in DILI with deranged clotting.^36^ A short trial of NAC is therefore reasonable but should be stopped quickly if there is no response.

MARS or plasmapheresis effects an expected biochemical and clinical response^14 16 22^ but is not widely available, has a high resource utilisation and is associated with its own risks.

AKI commonly complicates AAS DILI. In this series, eight patients underwent renal biopsy; two had acute tubular necrosis,^17 18^ one had IgA nephropathy^37^ and five had bile acid nephropathy.^9^ In keeping with these histological findings, peak bilirubin correlates with peak Cr and a level of ~440 μmol/L predicts the development of AKI.^5^ For our patients, Cr mirrored the bilirubin once the bilirubin rose above ~440 μmol/L (figure 2A, B).

Lastly, our cases illustrate the importance of careful history-taking. Both patients initially denied taking AAS only admitting so on subsequent questioning. Reasons that patients may not report AAS use include embarrassment or fear of the legal implications. Under UK law, AAS are classified as class C drugs that can only be prescribed. They are, however, legal to import in person for personal use but not using postal or courier services. Under US law, AAS fall under schedule III where a medical certificate is required even for possession. Of additional concern, some AAS preparations, including ‘Megavol’ taken by patient 1, claim to have the benefit of liver protection by including NAC and milk thistle within the formulation!

In conclusion, AAS use is widespread and rising and all physicians are likely to encounter patients with AAS DILI. The updated RUCAM score and causality assessment should be calculated on all patients where DILI is suspected, although conclusively identifying AAS as the culprit agent can be challenging due to the frequent concurrent consumption of body building supplements and the clinical desire to give treatment. Thankfully, the prognosis is excellent and that, although there is a paucity of high-quality data to guide management, it is reasonable to consider antihistamines or UDCA in symptomatic patients or corticosteroids in those with extreme elevations of bilirubin associated with elevated Cr.
