# Supplementary material for: Androgenic anabolic steroid-induced liver injury: two case reports assessed for causality by the updated Roussel Uclaf Causality Assessment Method (RUCAM) score and a comprehensive review of the literature
Source: BMJ Open Gastroenterol. 2020 Nov 19;7(1):e000549. doi: 10.1136/bmjgast-2020-000549 (PMC7678230; doi:10.1136/bmjgast-2020-000549)
Supplement: Supplementary data [file bmjgast-2020-000549supp001.pdf]

BMJ Publishing Group Limited (BMJ) disclaims all liability and responsibility arising from any reliance placed on this supplemental material which has been supplied by the author(s)

Supplemental Table 1: Clinical and biochemical data for all included cases

	**Case no.**	**Country**	**Age**	**Sex**	**AAS taken**	**Clinical presentation**	**Time from starting AAS to presentation (days)**	**Time from stopping AAS to peak Bili (Days)**	**R Value**	**Pattern of liver injury**	**Updated RUCAM score & Causality grading**	**Peak Bili (umol/L)**	**peak ALT (IU/L)**	**Peak ALP (IU/L)**	**Peak Cr (umol/L)**	**Time to resolution from peak Bili (days)**	**Primary therapy**	**Other therapies**
Slater SD, 1976[1]	1	UK	62	M	Stanozol	Jaundice and pruritis, weight loss	210		0.92	cholestatic or mixed	6-Probable	156	51			105	Nil	
Everly RS, 1987[2]	2	UK	65	F	Stanozol	Jaundice and pruritis	120		1.48	cholestatic or mixed	6-Probable	660	140	284	404	135	Nil	
Everly RS, 1987[2]	3	UK	62	F	Stanozol	Jaundice and pruritis	150		0.31	cholestatic or mixed	6-Probable	732		534	133	135	Nil	
Everly RS, 1987[2]	4	UK	65	M	Stanozol	Jaundice and pruritis	40		0.58	cholestatic or mixed	7-Probable	798		360	1021	135	Nil	
Awaii HI, 2014[3]	5	US	18	M	Beastrodol 40mg	jaundice, vomiting, difficulty concentrating	14	28	7.31	Hepatocellular	6-Probable	658	229	218		90	UDCA	Colestyramine, Rifampicin
Brazeau MJ, 2015[4]	6	US	26	M	Mutant Plexx, Methylstenbolone, Epistan, Halodrol, 13-ethyl-3-methoxy-gona-2,5(10)-diene-17-one (Max LMG).	Jaundice and pruritis	120	14	5.58	Hepatocellular	3-Possible	84	223			90	nil	
Cabb E, 2016[5]	7	US	45	M	Oxandrolone 50mg OD and testoterone inj weekly	Jaundice and pruritis	104	25	1.85	cholestatic or mixed	3-Possible	109	162	286			nil	
Diaz FC, 2016[6]	8	Spain	29	M	Epithiostanol	Jaundice and pruritis	30	30	4.30	cholestatic or mixed	6-8Probable	1060	169	248	177	120	MARS	Standard medical therapy
Diaz FC, 2016[6]	9	Spain	20	M	Epithiostanol	Jaundice and pruritis	33	30	5.43	Hepatocellular	6-Probable	968	344	292	203	120	MARS	Standard medical therapy
Diaz FC, 2016[6]	10	Spain	23	M	Epithiostanol	Jaundice and pruritis	31	30	1.36	cholestatic or mixed	6-Probable	860	109	248		120	MARS	Standard medical therapy
Diaz FC, 2016[6]	11	Spain	26	M	Epithiostanol	Jaundice and pruritis	30	30	2.59	cholestatic or mixed	6-Probable	694	300	347	124	120	MARS	Standard medical therapy
Ding NS, 2013[7]	12	Australia	46	M	Stanozol 40mg, Methandrostenal-one 40mg	Jaundice and pruritis	210		1.27	cholestatic or mixed	3-Possible	302	125	295		49	UDCA	
Eapen J, 2018[8]	13	US	26	M	methyl-1-etiochoenolol-epietiocholanolo-ne	Jaundice and pruritis		14	2.65	cholestatic or mixed	4-Possible	616	106			60	MARS	Standard medical therapy
El Sherrif Y, 2013[9]	14	UK	25	M	Methyldrostanol-one	jaundice, lethargy anorexia	49	42	4.53	cholestatic or mixed	4-Possible	614	207	137		105	nil	
El Sherrif Y, 2013[9]	15	UK	45	M	Methyldrostanol-one	Jaundice and pruritis abdo pain	56	63	1.76	cholestatic or mixed	5-Possible	229	152	259	173		nil	
Elsharkawy AM, 2012[10]	16	UK	32	M	Methandrosteno-lone 5mg	Jaundice, nausea and vomitting	60	4	0.87	cholestatic or mixed	6-Probable	800	76	262		90	UDCA	Chlorphenamin and Colestyramine
Elsharkawy AM, 2012[10]	17	UK	16	M	Methandrostenol-one 10mg TDS	Jaundice, pruritis, abdo pain, nausea	27	7	2.31	cholestatic or mixed	6-Probable	635	156	203		120	UDCA	colestyramine, Rifampicin, naltrexone
Jasiurkowski B, 2006[11]	18	US	23	M	Methasterone (Superdrol) 10mg then 20mg	Jaundice, pruritis, abdo pain, nausea and vomitting	74	28	1.25	cholestatic or mixed	6-Probable	718	93	280	301	75	UDCA 1.2g	Hydroxyzine

Kafrouni MI, 2007[12]	19	US	40	M	Methasterone (Superdrol) 20mg	Jaundice pruritis and weight loss	110	42	2.17	cholestatic or mixed	4-Possible	855	301	416			Predniso-lone 40mg - 1 week, UDCA for pruritis	
Kafrouni MI, 2007[12]	20	US	31	M	Methasterone (Superdrol) and Halodrol	Jaundice, weakness and flu like Sx, Pruritis and weight loss	56	35	0.47	cholestatic or mixed	6-Probable	747	59	375		105	UDCA	colestyramine, hydroxyzine, rifampicin
Anand JS, 2006[13]	21	Poland	21	M	Trenbolone	Jaundice and pruritis	49		3.18	cholestatic or mixed	5-Possible	1120	106			60	UDCA but then MARS	Topical steroids, phenobarbital
Krishnan PV, 2009[14]	22	US	21	M	Methasterone (Superdrol)	Jaundice, pruritis, nauseas and anorexia		21	3.07	cholestatic or mixed	5-Possible	665	256			90	Prednisolone 40mg	
Krishnan PV, 2009[14]	23	US	30	M	Dehydroepiandrosteron e	Jaundice and pruritis	35		5.77	Hepatocellular	3-Possible	137	200			90		Hydroxyzine and colestyramine
Krishnan PV, 2009[14]	24	US	38	M	17a-methyl-etioallocho-lan-2-ene-17b-01	Jaundice, pruritis lethargy, nausea and weight loss	98	53	2.84	cholestatic or mixed	3-Possible	920	467		212	75	UDCA then Prednisolone 40mg added 3 weeks later for 'unremitting jaundice'	Hydroxyzine
Magee CD, 2016[15]	25	US	29	M	Methasterone, Stenbolone, desoxymethyltest-osterone	Jaundice and fatigue	70	10	3.33	cholestatic or mixed	6-Probable	340	112	101		56		
Hymel BM, 2013[16]	26	US	26	M	Mastabol (17 beta-Hydroxy-2alpha-methyl-5alpha-androstan-3- one propionate)	Jaundice, pruritis weight loss	41		0.80	cholestatic or mixed	6-Probable	404	117	441				Phenobarbitol
NASR J, 2009[17]	27	US	42	M	Methasterone (Superdrol)	Jaundice, pruritis weight loss	84		0.83	cholestatic or mixed	5-Possible	705	98	353	318	120	UDCA 1.2g	Hydorxyzine, naltrexone
Rosenfeld GA, 2011[18]	28	Canada	50	M	Methandrosteno-lone 10mg BD 3 weeks, TDS 5 weeks	Jaundice , abdo pain, weight loss	56	6	1.09	cholestatic or mixed	5-Possible	937	64	206	299			Standard medical therapy
Yoshida EM, 1994[19]	29	Canada	26	M	Stanazol 125mg im twice weekly	Jaundice, nausea and vomitting, malaise	56	28	0.72	cholestatic or mixed	4-Possible	876		369	418	150		Standard medical therapy
Shah NL, 2008[20]	30	US	33	M	Methasterone	Jaundice, pruritis, loss of appetite, amalaise		39	0.23	cholestatic or mixed	3-Possible	790	102	474	203	120	nil	
Shah NL, 2008[20]	31	US	28	M	Methasterone	Jaundice, pruritis lethargy abdo pain	120	30	1.62	cholestatic or mixed	7-Probable	872	99	217	407	60	nil	
Shah NL, 2008[20]	32	US	25	M	Methasterone	Jaundice, pruritis, lethargy, abdo pain, diarrhoea	42	30	1.72	cholestatic or mixed	5-Possible	599	289	182		60	nil	
[2] Shah NL, 2008[20]	33	US	20	M	Methasterone	Jaundice pruritis, malaise, anorexia constipation	42	32	4.27	cholestatic or mixed	6-Probable	547	554	184		60	nil	

Shah NL, 2008[20]	34	US	21	M	Methasterone	Jaundice, abdo pain, weight loss, anorexia, malaise	42	28	2.29	cholestatic or mixed	5-Possible		58	170		105	nil	
Sanchez-Osorio M, 2008[21]	35	Spain	29	M	Mesterolone, testosterone udecanoate, nandrolone, oxymethoone, stanazol, testosterone	Jaundice, pruritis, abdo pain, weight loss	105	5	1.02	cholestatic or mixed	8-Probable	581	54	694		150	UDCA	
Singh V, 2009[22]	36	US	51	M	Methasterone (Superdrol) dehydroepiandros-terone,	Jaundice	42	35	29.01	Hepatocellular	4-Possible	872	172	106		150	UDCA	Colestyramine, UDCA, Hydroxyzine, Plasmaphoresis
Singh V, 2009[22]	37	US	25	M	Methasterone (Superdrol) 2 pills per day for 2 months, Aminovol, ergomax	Jaundice and pruritis	60	28	6.38	Hepatocellular	5-Possible	923	236	111		60	nil	Zolpidem, doxepin, Colestyramine, Rifampicin
Singh V, 2009[22]	38	US	33	M	Methasterone (Superdrol)10mg TDS for 3 weeks	Jaundice and pruritis	35	14	1.73	cholestatic or mixed	6-Probable		69			120	nil	
Stepien PM, 2009[23]	39	Poland	19	M	Stanazol 50mg im alt days	Jaundice, pruritis, malaise	60	21	2.45	cholestatic or mixed	6-Probable		135	77	141	135	initially UDCA and LOLA then due to failure hydrocor-tisone.	
Wree A, 2011[24]	40	Germany	46	M	?				0.00	cholestatic or mixed	4-Possible	547	66	403			UDCA 750 and Pred 50mg 3d	
Wree A, 2011[24]	41	Germany	22	M	?				0.00	cholestatic or mixed	4-Possible	410	52				UDCA 1500, Pred 1g 3d	
Wree A, 2011[24]	42	Germany	22	M	?				0.00	cholestatic or mixed	4-Possible	650	67				UDCA 1500, Pred 1g 3d	
Wree A, 2011[24]	43	Germany	24	M	?				0.00	cholestatic or mixed	4-Possible	1163	53				UDCA 750, Pred 250mg 3d	
Wree A, 2011[24]	44	Germany	26	M	?				0.00	cholestatic or mixed	4-Possible	1180	59				UDCA 750, Pred 250mg 3d	
Flores A, 2016[25]	45	US	31	M	Promagnon and Epitiostanoll	Pruritis, jaundice, AKI mild confusion			0.00	cholestatic or mixed	3-Possible	906						Hydroxyzine, Colestyramine, UDCA, sertraline, naloxone
El Khoury, 2017[26]	46	Lebanon	35	M	Stanazol, Trenbolone, testosteroine proprionate	Jaundice, Pruritis, Abdo pain and vomiting			8.28	Hepatocellular	3-Possible	549	450	329			UDCA	Hydroxyzine
Luciano RL, 2014[27]	47	US	41	M	Nandrolone, Testosterone, methandrostenol-one	Jaudice, Pruritis, malaise, weight loss		52	6.68	Hepatocellular	2-Unlikley	819	267				UDCA	Hydroxyzine, Colestyramine
Tabatabaee SM, 2015[28]	48	Iran	30	M	Stanazol	Jaundice, nausea, malaise		0	0.19	cholestatic or mixed	4-Possible	821	33	535			nil specified	Haemodialysis
Tabatabaee SM, 2015[28]	49	Iran	43	M	Stanazol	Jaundice		36	0.29	cholestatic or mixed	4-Possible	992	66	690				Haemodialysis

Viella AL, 2013[29]	50	US	50	M	Incredible Bulk and Spartan 45(α- methyl-etioallocholan-2-ene-17β-ol 15 mg, 2α,3α-epithio- 17α-methyletioallocho-lanol 10 mg, and Bulgarian Tribulus Terrestris extract 300 mg; Spartan 45 : 4-chloro-17α-methyl-androst-4-ene-3,17β-diol 15 mg, 2α, METHASTERONE 17α-dimethyl-17β-hydroxy-5α-androstan-3-one 15 mg, and 13-ethyl-3-methoxy-gona-2,5(10)-diene-17-one)	Jaundice, diarrhoea	60	9	2.34	cholestatic or mixed	4-Possible	535	125	180		105	Prednisolone short course then UDCA added	Colestyramine, Hydroxyzine
Abeles RD	51	UK	30	M	Superdrol	Jaundice, malaise, diarrhoea	42	21	11.68	Hepatocellular	4-Possible	633	257	123	122	90	Prednisolone 60mg then 40mg	
Abeles RD	52	UK	39	M	Methandienone (Xyenobol Dianabol10mg)	Jaundice, abdo pain pruritis malaise	56	53	6.30	Hepatocellular	4-Possible	768	460	603	166	76	NAC intitally for 5 days, no response, UDCA 750 no response. Pred 40mg started respnse but loss then regained	Antihistamines
**Summary stats**	**N**	**Region**	**Age**	**Sex**	**AAS taken**	**Clinical presentation**	**Time from starting AAS to presentation (days)**	**Time from stopping AAS to peak Bili (Days)**	**R Value**	**Pattern of liver injury**	**Updated RUCAM score & Causality grading**	**Peak Bili (umol/L)**	**peak ALT (IU/L)**	**Peak ALP (IU/L)**	**Peak Cr umol/L**	**Time to resolution from peak Bili (days)**	**Primary therapy**	**Other therapies**
Median	52 cases	North America 23 (44%) Europe 25 (48%) Mid east 3 (6%) Australia 1 (2%)	29	Male 50 (96%)			56	28	2.0	Hepatocellular 10 (19%) cholestatic or mixed 42 (81%)	Median 5.0 Unlikely 1 (2%) Possible 31 (60%) Probable 20 (38%)	705	125	190	203	90		
25th Percentile			24				41.5	14	0.8		4.0	549	71	123	167	75		
75th percentile			38				72	35	4.3		6.0	872	234	287	300	120		

AAS Andorgenic Anabolic Steroid, Bili Bilirubin, ALT Alanine Amino-Transferase, ALP Alkaline Phosphatase, Cr Creatinine, UDCA Ursodeoxycholic Acid, MARS Molecular Adsorbent Recirculating System, LOLA L-Ornithine L-Aspartate, NAC N-Acetyl Cisteine.

R Value calculated (ALT/ULN ALT)/(ALP/ULN ALP), if no ALP available then assumed to be 1. RUCAM Roussel Uclaf Causality Assessment Method

1. Slater SD, Davidson JF, Patrick RS. Jaundice induced by stanozolol hypersensitivity. Postgrad Med J 1976;52:229-32.
2. Evely RS, Triger DR, Milnes JP, Low-Beer TS, Williams R. Severe cholestasis associated with stanozolol. Br Med J (Clin Res Ed) 1987;294:612-3.
3. Awai HI, Yu EL, Ellis LS, Schwimmer JB. Liver toxicity of anabolic androgenic steroid use in an adolescent with nonalcoholic fatty liver disease. J Pediatr Gastroenterol Nutr 2014;59:e32-3.
4. Brazeau MJ, Castaneda JL, Huitron SS, Wang J. A Case Report of Supplement-Induced Hepatitis in an Active Duty Service Member. Mil Med 2015;180:e844-6.
5. Cabb E, Baltar S, Powers DW, Mohan K, Martinez A, Pitts E. The Diagnosis and Manifestations of Liver Injury Secondary to Off-Label Androgenic Anabolic Steroid Use. Case Rep Gastroenterol 2016;10:499-505.
6. Diaz FC, Saez-Gonzalez E, Benlloch S, et al. Albumin dialysis with MARS for the treatment of anabolic steroid-induced cholestasis. Ann Hepatol 2016;15:939-43.
7. Ding NS, De Cruz P, Lim L, Thompson A, Desmond P. Androgenic-anabolic steroid drug-induced liver injury. Intern Med J 2013;43:215-6.
8. Eapen J, Ayoola R, Subramanian RM. 'The efficacy of extracorporeal liver support with molecular adsorbent recirculating system in severe drug-induced liver injury'. Oxf Med Case Reports 2018;2018:omx077.
9. El Sherrif Y, Potts JR, Howard MR, et al. Hepatotoxicity from anabolic androgenic steroids marketed as dietary supplements: contribution from ATP8B1/ABCB11 mutations? Liver Int 2013;33:1266-70.
10. Elsharkawy AM, McPherson S, Masson S, Burt AD, Dawson RT, Hudson M. Cholestasis secondary to anabolic steroid use in young men. BMJ 2012;344:e468.
11. Jasiurkowski B, Raj J, Wisinger D, Carlson R, Zou L, Nadir A. Cholestatic jaundice and IgA nephropathy induced by OTC muscle building agent superdrol. Am J Gastroenterol 2006;101:2659-62.
12. Kafrouni MI, Anders RA, Verma S. Hepatotoxicity associated with dietary supplements containing anabolic steroids. Clin Gastroenterol Hepatol 2007;5:809-12.
13. Anand JS, Chodorowski Z, Hajduk A, Waldman W. Cholestasis induced by parabolan successfully treated with the molecular adsorbent recirculating system. ASAIO J 2006;52:117-8.
14. Krishnan PV, Feng ZZ, Gordon SC. Prolonged intrahepatic cholestasis and renal failure secondary to anabolic androgenic steroid-enriched dietary supplements. J Clin Gastroenterol 2009;43:672-5.
15. Magee CD, Witte S, Kwok RM, Deuster PA. Mission Compromised? Drug-Induced Liver Injury From Prohormone Supplements Containing Anabolic-Androgenic Steroids in Two Deployed U.S. Service Members. Mil Med 2016;181:e1169-71.

16. Hymel BM, Victor DW, Alvarez L, Shores NJ, Balart LA. Mastabol induced acute cholestasis: A case report. World J Hepatol 2013;5:133-6.
17. Nasr J, Ahmad J. Severe cholestasis and renal failure associated with the use of the designer steroid Superdrol (methasteron): a case report and literature review. Dig Dis Sci 2009;54:1144-6.
18. Rosenfeld GA, Chang A, Poulin M, Kwan P, Yoshida E. Cholestatic jaundice, acute kidney injury and acute pancreatitis secondary to the recreational use of methandrostenolone: a case report. J Med Case Rep 2011;5:138.
19. Yoshida EM, Karim MA, Shaikh JF, Soos JG, Erb SR. At what price, glory? Severe cholestasis and acute renal failure in an athlete abusing stanozolol. CMAJ 1994;151:791-3.
20. Shah NL, Zacharias I, Khettry U, Afdhal N, Gordon FD. Methasteron-associated cholestatic liver injury: clinicopathologic findings in 5 cases. Clin Gastroenterol Hepatol 2008;6:255-8.
21. Sanchez-Osorio M, Duarte-Rojo A, Martinez-Benitez B, Torre A, Uribe M. Anabolic-androgenic steroids and liver injury. Liver Int 2008;28:278-82.
22. Singh V, Rudraraju M, Carey EJ, et al. Severe hepatotoxicity caused by a methasteron-containing performance-enhancing supplement. J Clin Gastroenterol 2009;43:287.
23. Stepien PM, Reczko K, Wieczorek A, et al. Severe intrahepatic cholestasis and liver failure after stanozolol usage - case report and review of the literature. Clin Exp Hepatol 2015;1:30-3.
24. Wree A, Dechene A, Herzer K, et al. Steroid and ursodesoxycholic Acid combination therapy in severe drug-induced liver injury. Digestion 2011;84:54-9.
25. Flores A, Nustas R, Nguyen HL, Rahimi RS. Severe Cholestasis and Bile Acid Nephropathy From Anabolic Steroids Successfully Treated With Plasmapheresis. ACG Case Rep J 2016;3:133-5.
26. El Khoury C, Sabbouh T, Farhat H, Ferzli A. Severe Cholestasis and Bile Cast Nephropathy Induced by Anabolic Steroids Successfully Treated with Plasma Exchange. Case Rep Med 2017;2017:4296474.
27. Luciano RL, Castano E, Moeckel G, Perazella MA. Bile acid nephropathy in a bodybuilder abusing an anabolic androgenic steroid. Am J Kidney Dis 2014;64:473-6.
28. Tabatabaee SM, Elahi R, Savaj S. Bile cast nephropathy due to cholestatic jaundice after using stanozolol in 2 amateur bodybuilders. Iran J Kidney Dis 2015;9:331-4.
29. Vilella AL, Limsuwat C, Williams DR, Seifert CF. Cholestatic jaundice as a result of combination designer supplement ingestion. Ann Pharmacother 2013;47:e33.
