# Supplementary material for: Androgenic anabolic steroid-induced liver injury: two case reports assessed for causality by the updated Roussel Uclaf Causality Assessment Method (RUCAM) score and a comprehensive review of the literature
Source: BMJ Open Gastroenterol. 2020 Nov 19;7(1):e000549. doi: 10.1136/bmjgast-2020-000549 (PMC7678230; doi:10.1136/bmjgast-2020-000549)
Supplement: Supplementary data [file bmjgast-2020-000549supp002.pdf]

Supplemental Table 2: Comparison of current series and the Spanish/South-American series of Robles-Diaz *et al.*

	Current series (Abeles RD)	Robles-Diaz [1]
Number of cases	52	25
Age	29	31
Male	50 (96%)	25 (100%)
Days from starting AAS to presentation	56	49
Bilirubin umol/L	314	241
ALT IU/L	125	784
ALP IU/L	190	191
Renal dysfunction	22 (43%)	6 (24%)

AAS Androgenic Anabolic Steroid, ALT Alanine transferase, ALP Alkaline Phosphatase

1. Robles-Diaz M, Gonzalez-Jimenez A, Medina-Caliz I, et al. Distinct phenotype of hepatotoxicity associated with illicit use of anabolic androgenic steroids. Aliment Pharmacol Ther 2015;41:116-25.
